# Values and Workplace Expectations to Facilitate Retention: Perspectives From Nurses at Two Ends of the Career Spectrum

**DOI:** 10.1155/jonm/9912825

**Published:** 2025-09-12

**Authors:** Ourega-Zoé Ejebu, Jaimie Ellis, Liying Sun, Jane Ball, Julia Philippou, Anne Marie Rafferty, Sophie Painter, Joanne Turnbull

**Affiliations:** ^1^School of Health Sciences, University of Southampton, Southampton, UK; ^2^School of Applied Social and Policy Sciences, Ulster University, Belfast, UK; ^3^Royal College of Nursing, London, UK; ^4^Florence Nightingale Faculty of Nursing, Midwifery & Palliative Care, King's College, London, UK

**Keywords:** early-career nurse, job expectations, job factors, late-career nurse, NHS, qualitative research, retention

## Abstract

**Background:** Nursing shortages are detrimental to healthcare services due to the loss of skills and experience in patient care. In England, the retention of NHS nurses in their early- and late-career stages is of particular interest because they have the highest leaver rates.

**Aim:** To explore in detail what early- and late-career NHS nurses value and expect from their employers to retain them in their jobs and the profession. Insights from nurses at two ends of the career spectrum could offer a new perspective and shed light on seemingly persistent and detrimental factors for retention.

**Methods:** Semistructured focus groups and interviews, using Microsoft Teams, were conducted between April 2023 and February 2024 with early-career nurses (i.e., first registration between 2019 and 2024) and late-career nurses (i.e., 55 years and over). We also analysed open-text comments from a related survey (2023), which explored nurses' working lives and retention.

**Results:** 27 nurses participated in the qualitative study, and 784 nurses provided open-text comments from the cross-sectional survey. Except for tailored support when entering the profession and adequate remuneration when leaving nursing, the organisational factors cited by nurses as key to their retention were similar for both groups. Some of these ‘persistent' factors potentially detrimental to retention across both groups included a negative work culture, lack of adequate resources and limited opportunities for career development. Perceived inadequate remuneration should not be underestimated either.

**Conclusion:** Support from the leadership team and/or colleagues seems to partially alleviate the stress of working in strenuous environments. However, addressing ‘persistent' and detrimental organisational factors throughout nurses' careers should continue to be a priority to retain them, safeguard their well-being and enable them to deliver the standard of care they aspire to.

**Nursing Management Implications:** The findings have significant implications for improving the retention of early- and late-career NHS nurses.

## 1. Introduction

In many countries, registered nurses represent the largest proportion of the health workforce and play a pivotal role in the delivery of healthcare [1]. Yet, the difficulties in recruiting and retaining nurses have persisted over time, exacerbating global shortages and threatening the effectiveness of healthcare services [2–4]. The World Health Organisation (WHO) predicts an estimated global shortfall of 4.5 million nurses by 2030 [5]. In England, the gap between nurse supply and demand in the National Health Service (NHS) is predicted to reach 30,300 by 2030/31 [6].

In England, NHS nurses in their early-career (EC) (defined here as less than 5 years of work experience) and late-career (LC) stages (defined as nurses over 55 years) have the highest leaver rates in comparison with other groups [7–9]. This represents a strain on the labour market because the loss of recently qualified nurses causes a gap in the current year and a potential loss of 30+ years of future service. Related to this, an analysis of staff records from acute NHS Trusts in England shows that older nurses are more likely to leave [7–9]. The loss of experienced nurses nearing retirement also represents a potential loss of future services, albeit for a shorter period (e.g., a decade) compared to EC nurses. Most importantly, their departure depletes critical clinical knowledge, patient care expertise and mentorship for EC nurses, weakening workforce sustainability.

The lack of nurses in the workforce has consequences for both nurses and the delivery of healthcare. Heavy workloads and persistent staffing shortages compromise patient care quality by leaving fundamental tasks unfinished, heightening the risk of adverse events and contributing to longer patient waiting times [10, 11]. In turn, work pressure may induce nurses to compromise patient care and increase the risk of departure [12]. The resulting strain on the remaining staff often leads to reduced motivation, increased job dissatisfaction, heightened burnout levels and sickness absences—factors that further accelerate nurse turnover [13]. The unprecedented strike actions that occurred globally in the past few years, including in the United Kingdom (UK) [14], have highlighted that the nursing workforce is not only demanding higher wages, but is also expecting considerable improvements in their working environment to deliver patient care to the standard they aspire to [15].

Understanding in more detail what nurses at different career stages value and expect from their employers is crucial for retention and should be a priority for policymakers and healthcare leaders. These conditions could be referred to as ‘pull factors' as they encourage nurses to remain in their job or workplace. In contrast, ‘push factors' are conditions that drive nurses away from their profession [16, 17]. Previous research has highlighted that career progression, recognition and workplace conditions significantly impact nurse retention, reinforcing the need for targeted retention strategies across different stages of work [18]. For example, the long-awaited NHS long-term workforce plan [19] envisages that predictable career paths, flexible working patterns and improvement of the culture and leadership across organisations will contribute to enhancing nurse retention. Recognition of the push and pull factors associated with nurse retention is also captured in national policies, including the NHS People Promise [20], which focuses on the actions nurses (and healthcare staff) expect their employers to adopt to foster positive working environments most likely to retain them. Other policies, including the N50K Nurse Programme (i.e., Government initiative to increase the NHS nursing workforce by 50,000 by 2024) [21], have been implemented to enhance nurse recruitment and retention by focussing on flexible working patterns and improving work culture and leadership across organisations. Yet, it is unclear which of these promises and interventions are most important, how they are perceived and whether they are valued equally at all stages of the career span.

The push and pull factors associated with the retention of nurses have been widely studied in the international literature [22–24]. However, to design tailored retention strategies, a more nuanced approach would be beneficial to provide a detailed understanding of what nurses at different career stages value and expect from their employers to retain them in the profession. For example, a mixed-method study found that, for LC nurses in England, flexible work patterns are the strongest predictors of work beyond retirement [25]. However, the age range of participants varied from 21 to 70 years, suggesting that for many participants, retirement was a distant event. Study participants were also recruited from the same integrated care system (ICS)/geographical location, potentially limiting the generalisability of the findings. Another narrative review found that EC nurses differed in their expectations from nurses at other career stages, but most of the evidence stems from outside of the UK (notably the United States of America (USA), Canada and Australia) [16]. This prevents the application of evidence-based research to England and the NHS in particular.

To address these limitations, and acknowledge calls for more exploration and detailed qualitative work on this topic, our study uses qualitative methods to address the following research aim: to explore in more depth what NHS nurses in their EC and LC stages value and expect from their employers to stay in their jobs and the nursing profession. Comparison among nurses at two ends of a career spectrum provides a new perspective. It enabled us to capture differences and similarities in what nurses perceive as important to remain in their jobs and nursing, as well as capture seemingly persistent factors across career stages that may contribute to their intention to leave. This new knowledge can help inform strategies and policies to retain these groups of nurses.

## 2. Methods

### 2.1. Study Design

This exploratory qualitative study comprised focus groups, interviews and free-text responses from the cross-sectional survey of nurse experiences we conducted in 2023 [26]. The study is reported according to the Consolidated Criteria for Reporting Qualitative Studies (COREQ) checklist for qualitative research [27] (Supporting File 1). The qualitative data were collected as part of a larger study designed to evaluate the UK Government's initiative to increase the NHS nursing workforce in England by 50,000 (i.e., N50K Nurses Programme) [26, 28]. Focus groups and interviews were conducted by Ourega-Zoé Ejebu and Joanne Turnbull to capture a nuanced understanding of EC and LC nurses' experiences, as well as to inform the design of discrete choice experiments to elicit NHS nurses' preferences at EC and LC stages [22].

### 2.2. Recruitment

#### 2.2.1. Focus Groups and Interviews

We used several channels to recruit participants, including social media (X, formerly Twitter), our professional networks and the cross-sectional survey of nurses, which included a question where respondents could agree to be contacted for future research [26]. We purposively sampled nurses to represent different demographic characteristics and job roles and capture a diversity of experiences and views across participants. Recruitment was conducted until reaching data saturation, that is, until we were confident that we had collected sufficient data to yield enough information power to elucidate the aims of the study [29].

Participation in the online focus groups and interviews was completely voluntary. Nurses who indicated an interest in participating in the study were emailed an electronic participation information sheet (PIS) and a consent form by the researcher (Ourega-Zoé Ejebu). If they agreed to participate, they were asked to return the signed consent form to the researcher (Ourega-Zoé Ejebu). A signed consent form by both parties (researcher and participant) was then emailed back to the participants. Participants were given a £20 gift voucher following the completion of the interviews/focus groups as a token of appreciation for their participation.

#### 2.2.2. Cross-Sectional Survey

The cross-sectional survey was designed in 2023 by our research team to capture the recruitment and retention experiences of nurses working across NHS and non-NHS organisations [26]. It was distributed by the Nursing and Midwifery Council (NMC) on behalf of the research team to a random sample of 45,000 registered nurses in England. The sample included those with dual qualifications (e.g., nurses and midwives). 3048 nurses responded to the survey, corresponding to a 6% response rate (as anticipated by the NMC). There was no financial incentive for participating in the survey.

### 2.3. Study Participants

The participants in the qualitative study were all registered nurses working in NHS England at the time of the study.

Focus groups and interview participants: EC nurses were defined as those with less than five years of work experience, specifically nurses who first registered with the NMC between 2018 and 2023 at the time of the study (i.e., 2023). LC nurses were defined as individuals 55 years and over, which is in line with other studies' definitions of older workers [30–33]. We also posit that in the UK context, retirement may be a distal event for those aged 55 and under, where, at the time of data collection, the minimum eligibility for a state pension was 65. Nurses not meeting these criteria were not included in the qualitative study.

Cross-sectional survey participants: EC nurses were defined similarly to the focus group and interview participants (i.e., individuals who first registered with the NMC between 2018 and 2023). LC nurses were defined as individuals 51 years and over. The slight age difference between the focus groups and the survey stems from the NMC age grouping that we followed when designing the cross-sectional survey.

### 2.4. Data Collection

#### 2.4.1. Focus Groups and Interviews

Eight semistructured focus groups and seven interviews were conducted and video-recorded online using Microsoft Teams between April 2023 and February 2024 (Ourega-Zoé Ejebu and Joanne Turnbull). Interviews were offered to participants who were not available to attend the focus groups. Focus groups and interviews were conducted separately for EC and LC nurses. Focus groups were conducted once and did not exceed 1 h. Likewise, interviews were conducted once and did not exceed 1 h. Field notes were made during the focus groups and interviews to capture contextual information. No further contact was made by the research team with participants following the focus groups and interviews.

The semistructured discussion guides comprised questions to explore what nurses valued, what they expected from their employers/workplace and how these may influence their decisions to remain in their jobs and the nursing profession. Some questions were specifically tailored to nurses' career stages, such as questions on organisational support at the start of their careers (i.e., preceptorship and mentoring) and questions on retirement for LC nurses. The semistructured discussion guides were developed using evidence from the international and UK literature [16, 30, 34]. Insights from our Nurse, Patient and Public Involvement (NPPI) group also informed the development of the discussion guides. The NPPI group comprised a mix of EC and LC NHS nurses from different healthcare settings and diverse in terms of age, gender and ethnicity. In addition, feedback from our Study Advisory Group (SAG) was also used. The SAG included experts in nurse recruitment and retention policies (NHS England, NHSE and the Department of Health and Social Care, DHSC), professional bodies (Nursing and Midwifery Council, NMC) and a range of academics with expertise in labour market decisions. The semistructured discussion guides included key topics and prompts from the researchers to probe for a deeper understanding of participants' views (Supporting File 2).

The researchers leading the focus groups and interviews (Ourega-Zoé Ejebu and Joanne Turnbull) were female academics with extensive experience in leading qualitative studies, and none were related to the participants. Both researchers strived to create a welcoming and nonthreatening online environment for participants to share their work experiences. They encouraged the participants' equal participation during the focus groups to ensure the power relations were balanced [35]. Furthermore, the questions asked during the focus groups and interviews were iterative and continued until the participants felt they had sufficiently shared their experiences.

#### 2.4.2. Cross-Sectional Survey

Open-ended text data from the cross-sectional survey, designed to capture the qualitative recruitment and retention experiences of nurses, are also included in this study [26]. Specifically, the survey included an open-text statement that read ‘If there is anything you'd like to add about your experience of working as a nurse, or recruitment and retention in the NHS, please use this space: (open-ended)'. The responses to this statement from nurses working in the NHS were collated and analysed alongside the interview and focus group data.

### 2.5. Ethics Approval

We obtained approval from the Ethics and Research Governance Online (ERGO) (University of Southampton) committees to conduct this study (ERGO # 8061.A2).

## 3. Data Management and Analysis

The transcripts from the recorded focus groups and interviews were safely stored (the University of Southampton iSolutions secure Research Filestore service), anonymised and independently checked by the team members for accuracy (Ourega-Zoé Ejebu, Joanne Turnbull and Sophie Painter). Transcribed focus groups, interviews and cross-sectional survey data were entered into the analysis software NVivo Pro Version 12 [36] to facilitate data management and multianalysis data coding and enhance rigour. We used a thematic analytical approach, broadly following the stages described by Braun and Clarke [37].

For each nurse group, the research team (Ourega-Zoé Ejebu, Joanne Turnbull, Liying Sun and Jaimie Ellis) initially read and open-coded a sample of transcripts to develop emergent codes and themes, which were refined and applied to all transcripts. Coding and interpretation occurred iteratively and inductively, focussing on the research questions. We used constant comparison to identify and develop codes that were common across both groups, as well as those that differed. We coded these to provide new insights into the push–pull factors among nurses at two ends of the career spectrum. The team interpreted the data to build emergent themes and develop narrative and interpretive summaries.

We report verbatim quotes, identifying participants by career stage (i.e., EC and LC) and indicate if the participants were from the cross-sectional survey (S) or the focus groups/interviews (F). Where less relevant text has been removed, this is indicated by an ellipsis (…). Any nonverbatim explanatory text is in square brackets.

## 4. Results

### 4.1. Description of the Sample

#### 4.1.1. Focus Groups and Interview Participants

In total, 27 registered NHS nurses participated in the qualitative study. More specifically, this included 11 EC nurses (*n* = 6 participated in three focus groups) with a mean of 53 min and 42 min for the focus groups and interviews, respectively. 16 LC nurses took part in the qualitative study (*n* = 14 participated in five focus groups), with a mean of 55 min and 60 min for the focus groups and interviews, respectively.  Table 1 shows the summary of the interviewees' details for each group of nurses. For each group, there was a diversity of nurses in terms of geographical region, field of nursing and age. However, participants were predominantly White and female.

#### 4.1.2. Cross-Sectional Survey Participants

In total, 166 and 1423 were defined as EC and LC nurses, respectively. Among these, 56 EC (34%) and 728 LC (51%) NHS nurses completed the free-text box, whose responses were subsequently analysed. There is a diversity in terms of field of nursing and geographical regions. The participants were predominantly White and female, aligning with the NHS composition of the nursing workforce in England [38]. See Table 2 for demographic information about the whole sample.

### 4.2. Findings

We identified factors relating to retention that operate at the macro-, meso- and microlevels. Push–pull factors were identified, including the work environment, the government and personal fulfilment. These factors were categorised into two main overarching themes:

• Valuing nursing (systemic and professional factors). This theme centres on nurses' perceptions of their roles and profession, workplace conditions and the overall standards of healthcare delivery.
• Valuing nurses (individual expectations). This theme focuses on individual-centred factors (e.g., career development, professional recognition and personal well-being) that EC and LC nurses expect to stay, be motivated and develop in their profession.

Across the two groups, consensus existed on several factors and some disparity existed on others. These consensuses and disparities are drawn out throughout the results.

#### 4.2.1. Theme 1: Valuing Nursing—the Nursing Profession and Work Environment

Both EC and LC nurses provided insights on how their job perception, perceived value from employers and policymakers, work environment and available patient care resources were associated with their retention, i.e., the decision to stay in, leave or return to their jobs and/or the nursing profession. The next subsections provide more details.

##### 4.2.1.1. Valuing a Caring Profession: Making a Difference

Both groups valued their professional identity as nurses and the role they played in patients' recovery at a time when they were particularly vulnerable. For most, the vocational aspect of nursing was a defining feature for entering and staying in the profession. LC nurses did not want to stop working and perceived they still had a wealth of skills and expertise to offer patient care (even when they were physically impaired in some instances). Some chose to apply their skills to other roles within the NHS that were not frontline nursing roles. The desire to continue making a difference, sometimes in educational or leadership roles, played an important part in their decisions to delay retirement.

*F/EC nurse #2 “I think nurses see people at the very extremes of their life, when they're most in pain, when they're dying, when they're sick, and when they're scared. I love to be with people in those moments. That's what brought me to nursing in the first place.”*

*S/EC nurse #1 “Gaining and retaining nurses is reliant on people that want to help people as a job and there is no incentive to come to the job other than this.”*

*F/LC nurse #3 I feel that nursing is a privilege and continue to think so, particularly being a neonatal nurse, is a huge privilege. I spent probably a very long time in a senior clinical role in a very busy neonatal intensive unit thinking ‘Oh, I can't ever imagine not caring for the babies and their families.' But had a real interest in education […] I was suddenly […] in my early 50s, [thinking] I need to make a bigger impact.*

*F/LC nurse #4 “I feel like I haven't finished. I feel like there's still more to do, and I still enjoy what I do, and feel I have still got a lot to give with regard to the next couple of years or however long that takes.”*

*F/LC nurse #11 “I was a practice development sister. My situation was that I got long-Covid […]. The situation of working, doing practice development and trying to juggle with long-Covid: I just couldn't carry on. So that's why I ended up retiring […]. It wasn't my intention to retire when I did. It was just my situation [long Covid].”*

Both EC and LC nurses gained satisfaction from supporting their colleagues. They valued the perceived positive impact they had by supporting less experienced colleagues, but also offering wider support, and helping other nurses navigate a pressured environment. All were motivating factors to continue working in nursing.

*F/EC nurse #1 “I really like the impact that I have on the learner; catching up with them in practice and seeing how they getting on.”*

*F/LC nurse #2 “That's why it's so important to me, from the legacy mentoring bit, is to pass on the bit to look after these newbies, to help them overcome some of the stresses and difficulties that they're finding. Because I just think if we don't, we're going to see a very different health service.”*

##### 4.2.1.2. A Yearning to Be Valued

Whilst nurses are motivated by the difference they make to patients' lives, there was an expectation from nurses that they should be valued by the government and healthcare system leaders. Having their contributions valued or recognised was particularly important for LC nurses in their decisions to stay in or leave their jobs, and sometimes the profession. Government policies were often perceived as focussing on recruiting new nurses, rather than retaining current nurses. As an example, government policies leading to significant recruitment of new nurses—a major component of the N50K Nurses Programme [21], led some LC nurses to feel that their knowledge, expertise and skills were under-recognised and undervalued. In contrast, despite retention being a part of the N50K Nurses Programme, most participants did not perceive that their employers implemented specific strategies to retain them over the past few years (including the period during which the N50K Nurses Programme was instigated). The few who were aware of this programme were sceptical about its effectiveness in terms of recruiting and retaining domestically educated nurses, in comparison to the efforts made by their organisations to employ nurses from other countries. LC nurses, in particular, felt that demonstrable and visible efforts to retain experienced nurses were lacking in many organisations. Notwithstanding, nurses' decisions to continue working were independent of their employers' (perceived) efforts to retain them.

*S/LC nurse #1: “NHS Trust ought to focus on staff retention as this has not been successful in this organisation. Valuing staff is lacking in some workplaces after so much effort goes into recruitment.”*

*F/LC nurse #7: “There's more of a focus about recruiting people, rather than thinking about how do we retain people who are late in their career.”*

*F/LC nurse #1: “But at the moment in my trust, the international recruitment has a very big focus (…) So they've had all that investment, skill and experience and time and then they [international nurses] disappear.”*

*F/LC nurse #9: “I didn't notice any effort in trying to retain experienced nurses at all. No one really cared if you left, apart from my new boss. So the reason I got my new job after I retired was because my new boss heard I was retiring, and thought it was a ridiculous waste of my experience for me to go off.”*

*S/LC nurse #2: “Ward nurses are overworked and undervalued by the Government for years. I am glad to no longer work there, as when I did I was exhausted, both physically and mentally by the end of the day.”*

##### 4.2.1.3. Concerned About the Future of the Profession

Unique to LC nurses was a feeling that the role of nursing had changed, and they were no longer able to deliver the care they desired. Both changes in their role and work seem to be a push factor for them, as well as a concern for future generations.

*S/LC nurse #7: “As I get older, the physical demands of the job are harder to deal with. In the last 10 years, the demands of the job have increased significantly whilst the rewards have reduced. I would like to leave but cannot afford to retire for some time.”*

*S/LC nurse #8: “Nursing should be fulfilling, but it's not any longer, it's very hard work and demoralising more often than not. We used to have good days and very few bad days, now it's bad days and more bad days with the very occasional good day. Coming towards the end of my career in nursing, I would love to be able to recommend nursing to others, but I don't do that any longer as I have witnessed too many broken new nurses, this is upsetting to witness.”*

*S/LC nurse #9: “Nursing since 1986 - a lot has changed. [I] love my job but feel staffing does not let me do it properly. Too much data input (however this is important). Just find my work as DN [District Nurse] frustrating atm [at the moment]. Very near to retirement now but feel concerned for younger nurses.”*

##### 4.2.1.4. Supportive Work Environment: Teams, Management and Organisational Culture

A supportive professional network and organisational culture were recognised as part of a positive work environment. Both EC and LC nurses viewed this as a buffer against a challenging environment. Relationships with colleagues and teamwork were extensively discussed among both groups. Positive relationships with colleagues and a supportive team were viewed as key factors in decisions to stay in their jobs and the profession, and this was particularly so among EC nurses.

*F/EC nurse #1 “I think the times when I have really struggled, a few colleagues pull through a little bit and I've made a lot of friends (…) I definitely think colleagues have helped me stay.”*

*F/EC nurse #2 “If I could show up to work every day and know that my team were supportive and I had the things that I need to do my job, and I've been given every chance of working safely, I would do nursing for the rest of my life. I love it.”*

*S/LC nurse #3: “I have enjoyed most of my time in the NHS. But that has to do with the friends I have made and because I know we make a difference to people's lives.”*

Managers and leaders were key colleagues who influenced nurses' job satisfaction and, in turn, could influence the decision to leave or stay in their jobs and the profession. In both groups, management style and practices, including offering support and explicitly acknowledging (and valuing) nurses' contributions, encouraged decisions to stay in or ‘return to practice' in certain instances. EC nurses particularly valued managers who were visible, accessible and supportive. In contrast, some LC nurses perceived that the presence of too many managers meant there were not enough (financial) resources to expand the nursing workforce. Thus, these added work pressure and short staffing were factors negatively associated with retention.

*F/EC nurse #3 “Having a good relationship with your manager definitely encourages me to stay. Because even if I make a mistake, for example, I know that it'll be dealt with and I'll be supported.”*

*F/LC nurse #6 “My managers were really keen that they didn't want me to go. For me, that was kind of them valuing the role, valuing what I brought to the organisation and the work that I did (…) That made me think, I'll stay.”*

*F/LC nurse #12 “If I returned and had some young manager telling me what to do in a pretty cavalier sort of way, I think I'd be very distressed about it (…) that my wisdom and experience have been disregarded and that I was being kind of dragooned in an unnecessary way, I think that would be the worst thing.”*

*S/LC nurse #4: “Nursing is not what it used to be. Middle management [is] dangerous. In my eyes too many of them and usually all “groupies”. Management do what they want not always fairly. So when you see what goes on it dampens your spirit sometimes. I am grateful to be coming to the end of my career.”*

Related to positive relationships with colleagues and managers, both EC and LC nurses cited a supportive working environment as an influential factor in their decisions to remain in post and the profession. A culture of cohesion and collegiality was recognised as a pull factor. Nurses perceived that more efforts were needed to foster positive work environments and prevent nurses from leaving. This included addressing toxic environments, characterised by bullying, harassment, intimidation, blame culture and nurses feeling their contribution was undervalued or not recognised. Both EC and LC nurses highlighted the role of management and leadership in shaping the culture of an organisation and its potential link with retention.

*F/EC nurse #4 “For me, that's the main thing: a nice culture to work with.”*

*F/LC nurse #1 “(…) Widespread intimidation. That's been there for 9 years, and it peaks at times, drops at times. But I guess I've become very familiar with that behaviour: harassment, bullying, extensive bullying (…) But at no time have I ever considered escalating my retirement plans, but I can imagine the majority would.”*

*S/EC nurse #2: “Sadly, I have seen wonderful colleagues with tremendous experience unappreciated by senior management causing them to leave.”*

A few nurses discussed how mechanisms to foster positive well-being or simply account for their physical health were important in retaining them or would have brought them back into nursing. This was especially notable among a few EC nurses, where the presence of an occupational health department prevented them from leaving during intense work episodes, for example, during the COVID-19 pandemic.

*F/EC nurse #8: “We've got [Name] network, which was a massive plus during Covid time. [It] is our mental health and well-being page, where we can access resources (…). That was really, really good. Obviously, it came at a time when we all needed it and where we were struggling. I think for my Trust, they easily recognise areas where support is needed and help is needed.”*

*F/LC nurse #11: “Flexibility of shift pattern to accommodate, if I'm having what I call a ‘long-Covid day'. And accessibility as well, because of my mobility problems (…) For Trust to be adaptable. They say I couldn't carry on doing my practice development role. But within that role, there are so many other bits you can do as a sideline; something that would use some of the skills that I've got without just stopping [to work] completely.”*

##### 4.2.1.5. Lack of Human and Capital Resources: Can't Care for Patients From an Empty Cup

Both nurse groups perceived difficulties in delivering the standard of patient care they aspire to. This was attributed to a lack of human and capital resources, including inadequate staffing levels and skill mix, as well as a lack of (clinical) equipment. Several EC nurses stated this was important in their decisions to leave or stay in their organisation and nursing, and some expressed frustration at this deficit. Among LC nurses, there was a greater sense of resignation regarding the lack of adequate resources, suggesting it was simply the reality of the NHS. However, the rewarding nature of the job alleviated the frustrations of being under-resourced. Others expressed disillusionment with the level and quality of patient care and regretted their decision to enter into nursing.

*F/EC nurse #2 “If I leave, it will be because – too often – I get home at the end of the day and I know that I've not done well because it's been too busy. There's not been enough of us on the floor, I just haven't been able to do what I know I should be doing, and that's devastating. It's awful.”*

*F/LC nurse #10 “I think after all these years in the NHS, a bit like salaries, you just sort of get used to your pay. [You] get used to your conditions, get used to the fact that IT systems are completely hopeless, the fact you can't get bits of equipment because it's too expensive, that you run out of syringes because they can't get delivered (…) Loads of things at work are dreadful. But I keep going because I like doing my job. Those things wouldn't make me leave because I'm just used to them.”*

*S/LC nurse #5: “Staff morale is low. I enjoy my work and work with great colleagues, which is why I'm happy staying where I am at the moment, but it's always pressure and short-staffed.”*

*S/LC nurse #6: “I wish I had never become a nurse. I am so disappointed with the care the patients receive. […] I would leave if I could.”*

What is clear across both EC and LC nurses is a driving force to continue in a demanding and dynamic profession, the difference they make in the lives of patients and the role they play with colleagues. However, where these contributions cannot be achieved and are not valued, this has a detrimental effect on the desire to stay in their jobs and the profession.

#### 4.2.2. Theme 2: Valuing Nurses—Support and Reward

Support and reward were cited as work features that would encourage nurses to stay, develop and thrive in their jobs and the profession. These features were, for the most part, specific to nurses' career stage, as evidenced in the next result subsections.

##### 4.2.2.1. Structured Support for (New) Nurses

Both EC and LC nurses perceived that the transition into nursing was less difficult when a period of structured support (e.g., preceptorship and/or mentoring) or less formal support was available. For EC nurses specifically, a caring and supportive team, including preceptors and/or mentors, was identified as particularly beneficial in boosting their confidence and helping them continue to work in a demanding environment.

*F/EC nurse #9 “Within theatres we have our preceptorship, which is completely different to the preceptorship that's on offer for the rest of the hospital. For me, that was a massive win and an easy choice to have the support that is needed when you first start as a newly qualified nurse.”*

*F/EC nurse #7 “I've been there for about 13 months (…) and everyone is really, really supportive. We've had a winter that was difficult. But everyone's really supportive. I think that if I hadn't had that level of support and that level of care (…) it would have been really, really difficult for me.”*

LC nurses expressed concerns as they perceived that EC nurses received less support compared to the past, yet the work expectations were higher.

*S/LC nurse #10: “Working as a nurse for over 30 years has become much harder, much more is expected of you, newly qualified nurses are left to sink or swim.”*

*S/LC nurse #11: “Training is now poor, students not equipped with skills when they qualify, hence stress and inevitable decisions to find other jobs.”*

##### 4.2.2.2. Recognition of Expertise and Supporting Career Development

Among both groups, there was a clear expectation that nurses wanted to be recognised by their leadership team and organisation for their expertise and skills; this was particularly notable for LC nurses. Nurses desired to work autonomously and for their clinical judgement to be taken into consideration in decision-making. Some nurses described a sense of stagnation, such as doing routine tasks that did not use their skills and experience, or expressed frustration at their skills being mismatched with their duties and decided to leave the nursing profession as a result. In contrast, recognition and decision-making seemed to encourage EC nurses to stay in their jobs.

*F/LC nurse #5: “My skills are not valued (…) I'm the only person at the practice who's trained and confident to start somebody on insulin and titrate their insulin. They would rather I acted as a phlebotomist than I saw all these complex diabetics (…) It's boring and frustrating. I suppose if they lifted the boredom and got rid of the frustrations, I'd be happy to continue [instead of leaving nursing].”*

*S/LC nurse #12: “The Trust does not value me or my skills and knowledge base. I've returned as planned until I can secure work outside nursing. I've had enough.”*

*F/EC nurse #6: “One thing my Trust did well, is doing right now, is diversity championing. I recently joined as part of the cohort this is sitting on decision-making. So that's something that made me stay.”*

In both groups, there was a clear consensus that career growth, the mechanisms in place to enable development and retention, was intrinsically linked. Several EC nurses noted a lack of opportunities and structured support to enable career growth and avoid ‘stagnation'. The lack of career growth and the ‘boredom' that could result from it explained that a few interviewees were leaving their current organisation for another. LC nurses also had clear expectations in terms of career progression. Similarly, many perceived that their employing organisations were not proactive in offering training opportunities because of their career stage and/or age. Nonetheless, LC nurses were keen to demonstrate their leadership skills.

*F/EC nurse #10 “The main reason I've left or choosing to leave, is that I'm not challenged mentally. I'm bored, it's stagnant, it's stale. There's nowhere to go (…) I'm a dual field nurse (…) I'm completely underutilised.”*

*S/LC nurse #13: “Throughout my nursing career, I have seen major changes, but have always believed the key is training and education to ensure recruitment and retention is maintained. This is from student nurse training to continuous professional development.”*

*S/LC nurse #14: “NHS should be doing all it can to hold on to older nurses, including helping them to advance. Just because someone has reached an age milestone does not automatically mean they want to slow down.”*

##### 4.2.2.3. Flexible Working Patterns

Work–life balance was extensively discussed in both groups. The need for work–life balance and flexible working patterns conducive to achieving this was key to retention. Whilst work–life balance had different meanings among nurses, there was a unanimous desire to have working patterns that could more flexibly fit with nurses' individual preferences, circumstances and needs to achieve work–life balance. LC nurses suggested flexible working patterns were important to them to remain in nursing. Some participants cited the high pressure and workload in nursing, and a more flexible role helped to ameliorate the demands of working on wards or shifts. Some nurses valued hybrid work to enable them to combine nursing with caring responsibilities or long-term health conditions.

*F/EC nurse #5: “I really do value work-life balance. I was finding I was not getting that on the wards (…) On my days off, rather than having time to spend with my family and doing the things I enjoy, like physical exercise and all that, I just wanted to have a ‘sofa day'. I just thought that wasn't particularly healthy for myself or my family life. So there was another reason for looking at the health visiting role. It's more of a ‘nine-to-five/Monday-to-Friday' role which I think suits me better.”*

*S/LC nurse #15: “Internal rotation is not agreeable with family life and shift work should be far more flexible. Some staff prefer to work nights as it suits family life and childcare, where other nurses hate nights, struggle with them and have left nursing because of this.”*

*F/LC nurse #16 “The flexibility that Covid has brought…we suddenly had to work from home. We've retained lots of those ways of working. It actually makes it easier as you move forward and towards retirement.”*

Unique to LC nurses—who perceived that flexibility did not exist within the healthcare system—was the option to ‘retire and return' as a strategy to achieve their desired work–life balance and illustrates the link between work–life balance and retention. A few participants changed organisations to reduce their working hours after they decided to retire and return to work.

*S/LC nurse #16: “As I get older and look towards retiring completely, to be able to be flexible about the hours I work is huge – the only reason I have returned after flexible retirement.”*

##### 4.2.2.4. Remuneration Matters

Salary was perceived as one of the means employers and decision-makers could express the extent they value and recognise the contribution that nurses make. Both EC and LC nurses highlighted that their salary was not commensurate with their responsibilities and skill level. Some EC nurses suggested their starting salary (Band £5–£29,969) should be reviewed and ideally made equivalent to what Band 6 nurses earn (£37,338). Furthermore, as highly skilled professionals, having their expertise recognised constituted a pull retention factor (i.e., factors in the work environment that keep nurses in their job and/or workplace).

*F/EC nurse #11“I don't think I realized until I started doing that[nursing], just how low paid we actually are for what we do (…) I think it probably needs to be a lot more than that because we are quite skilled professionals (…) maybe about £35,000 to start, even more.”*

*S/EC nurse #3: “Nurses these days are constantly having to upskill themselves, yet with no improvement to our pay. The easiest way to improve retention would be to increase pay. We need our skill set to be better reflected.”*

Unique to LC nurses was the role of work pensions in their decision to stay (e.g., delay retirement, retire and return) or leave nursing. In some instances, the decision to continue working after retirement was linked to the fact that some could not ‘afford' to fully retire. Certain LC nurses were not confident that their pension would be financially adequate for retirement. The majority of LC nurses stated that a large ‘lump sum' of money would incentivise them to retire. In the UK, from the age of 55, people can usually take up to 25% of their pension fund without paying tax (a ‘tax-free lump' sum), but some participants were often unsure of their likely pension benefits.

*F/LC nurse #8 “I think I'm still working because I'm just a bit unsure about what retirement would mean for me in terms of stability and lifestyle. Will my pay pension be adequate?”*

*F/LC nurse #14 “If I got a massive lump sum tomorrow, out of nowhere, then I would probably think about fully retiring before I reached pension age.”*

In other instances, the decision to delay retirement or retire and return was related to the features of their pensions (e.g., maximum allowance, benefits). As an example, the possibility to continue working whilst drawing from their 1995 pensions (and reducing their hours in some instances) led several nurses to retire and return to work.

*F/LC nurse #4 “The reason I've picked July [to retire] is to do with my pension. My pension is very old. I'm no longer paying into it, but it still honours the final salary, the last 3 years, best of three (…) It's not necessarily gonna get a bigger wage. So it's going to get pulled and I plan to come back on a slightly reduced hour schedule.”*

*F/LC nurse #9 “Everybody was being switched from the 1995 [pension] to the new one. It didn't financially make sense to stay working [full-time], or not retire [and return] because of what they [the NHS] were doing with the pensions. It would have capped my lump sum and my pension.”*

There was a general consensus among both groups of nurses that valuing nurses and acknowledging their expectations would (or could) help to retain them. Slight nuances existed, but in general, where nurses had their expertise recognised, developmental opportunities offered, a balanced work–life and commensurate salaries, they were more inclined to remain in the profession.

## 5. Discussion

Retaining nurses in the NHS and safeguarding their well-being requires employers and policymakers to understand and address their needs and preferences, and importantly, recognise that these may vary by career stage [24]. Our findings suggest that many factors influencing retention are consistent across EC and LC nurses, particularly the importance of feeling recognised and valued, a theme echoed in earlier research emphasising the role of workplace conditions and career progression in nurse retention [18]. These ‘pull factors' should be well within the control of employers, as highlighted in previous research [34, 39–41].

Our study found that nurses strongly valued their profession: the sense of vocation and pride nurses have when entering the profession is still very much present in nurses later in their careers, which has also been found elsewhere [42–44]. The enduring dedication to patient care, despite challenging and resource-limited environments, suggests significant retention potential. Furthermore, support from the leadership team and/or colleagues appears to mitigate the stress of demanding work conditions, aligning with previous findings [45, 46]. However, perceived changes in the nursing role, inadequate staffing and increasing demand were viewed as jeopardising the standard of patient care, and often a key factor in decisions to leave the job or the nursing profession. This highlights the importance of continuing to address the lack of safe staffing levels and equipment to ensure the safe delivery of patient care that nurses aspire to.

Examining retention across the two ends of the career spectrum enabled us to identify seemingly ‘persistent' factors that may negatively impact nurses' decisions to remain in their jobs and the profession. In other words, the expectations nurses had when they joined the profession to stay and thrive were, in some instances, not realised for nurses who approached retirement. This was particularly true for the negative work culture, lack of recognition and inadequate resources (staffing levels and/or equipment) and limited opportunities for career development—challenges that have been consistently identified as critical determinants of nurse retention over the years [46, 47]. Notably, our study included nurses with different job intentions (or career pathways), including nurses who were about to leave their employers, the nursing profession and those willing, but unable to, return to practice due to reasons beyond their control (e.g., long-term health conditions). Their inclusion was useful in analysing retention factors from different perspectives, including fostering career growth, valuing expertise or adapting the working environment and/or schedule to account for long-term health conditions.

Addressing these workplace challenges is essential to mitigating the ‘leaky bucket' phenomenon [10, 11], in which newly recruited staff enter unsupportive environments that ultimately drive attrition. Encouragingly, national policies, such as the NHS People Promise [20], are being implemented and adopted to ensure workplaces embed a positive culture to recruit and retain healthcare staff. Furthermore, the NHS long-term workforce plan [19] aims to retain more staff by improving opportunities for career growth, boosting flexible working patterns, allowing for enhanced work–life balance and improving the culture and leadership across organisations. Many nurses in our study expressed concerns that recruitment efforts were prioritised over retention (e.g., N50K Nurse Programme), supporting the need for a more balanced approach. These persistent challenges are unlikely to be solved by a ‘one-size-fits-all' solution, but tailored and targeted approaches—including co-design—for different groups of nurses experiencing workforce challenges may be a valuable approach. Furthermore, ranking methods, such as discrete choice experiments, could be beneficial to elicit and target the job factors most valued for different groups of nurses and the extent to which each group of nurses need to be compensated for less attractive job features [22]. Future research should also prioritise the evaluation of these policies and interventions to assess their effectiveness in terms of retention of nurses at different career stages.

Preventing the departure of nurses is key, considering the competitiveness of the UK labour market and the myriad of options available to nurses, EC nurses in particular. Retaining LC nurses is also key, as the transfer of their skills, expertise and wisdom is not only an opportunity to use their leadership skills, but could also benefit less experienced nurses. Nurses may be incentivised to leave for other employing organisations, other non-nursing jobs or even countries. As an example, Australia has used large financial incentives to attract overseas and British nurses [48]. Whilst pecuniary incentives alone are unlikely to retain nurses, our study found that nurses' remuneration (salary and/or pension) was perceived as inadequate given their roles and responsibilities or insufficient to face increasing living costs.

The strength of our study is its focus on retention factors among nurses at different stages of their careers. For each group, there was also a diversity of nurses in terms of geographical region, field of nursing and age. The limitations are that nurses working in settings outside the NHS were excluded from the study, so the findings may not be transferable to non-NHS sectors. Participants were predominantly White and female, suggesting that the perspective of nurses from Black and Minority Ethnic (BME) nurses may not be well represented in the data. Notably, a few LC nurses near retirement age had less than 5 years of nursing work experience, which slightly overlapped with our definition of EC nurses. Considering their age and their previous (non-nursing) work experience, such nurses were not considered as EC nurses in our study. Finally, the results presented in this paper are part of a larger study which convened the interviews and focus groups primarily intending to inform a discrete choice experiment [22], which influenced the nature of the semistructured discussion guide and the focus of the data collection.

## 6. Conclusion

Exploring what could retain nurses who are at two ends of a career spectrum provided new insights and enabled us to identify seemingly ‘persistent' factors. A particular concern is the fact that the expectations that new nurses have when they join the profession to stay and thrive are, in some instances, not realised for nurses who approach retirement. Addressing seemingly ‘persistent' and detrimental organisational factors throughout nurses' careers should continue to be a priority to retain them, safeguard their well-being and enable them to deliver the standard of care they aspire to.

## Figures and Tables

**Table 1:** Sociodemographic information of early- and late-career NHS nurses participating in the focus groups and interviews.

Career stage	Date of 1^st^ registration with the NMC	Age^∗^ [range]	Gender	Ethnicity	Work pattern	Managerial position	Region	Field of practice
Early-career	[2018–2023]	[23–50]	Female = 10 Male = 1	White = 9 BME^∗∗^ = 2	Full-time = 10 Part-time = 1 Retired = 0	Yes = 3 No = 8	London = 2 Midlands = 1 South East = 2 South West = 3 Yorkshire = 3	Adult care = 2 Community care = 2 Education and practice development = 2 Mental health = 2 Other = 1 Paediatric care = 2

Late-career	Not relevant	[55–77]	Female = 13 Male = 3	White = 14 BME = 2	Full-time = 9 Part-time = 5 Retired = 2	Yes = 9 No = 5 Not applicable = 2	London = 3 Midlands = 3 North East = 3 North West = 1 South East = 3 South West = 3	Adult care = 2 Community care = 3 Digital health = 1 Education and practice development = 1 Learning disability = 1 Mental health = 2 Older people's care = 1 Other = 4 Paediatric care = 1

^∗^Age is presented by range rather than using the mean, as some participants preferred not to reveal their age.

^∗∗^Black and Minority Ethnic (BME).

**Table 2:** Sociodemographic information of early- and late-career nurses participating in the cross-sectional survey.

Career stage	Date of 1^st^ registration with the NMC	Age [range]	Gender	Ethnicity	Work pattern	Region	Field of practice
Early-career	[2018–2023]	[21–70]	Female = 151 Male = 14 PNTS = 1	White = 122 BME = 41 PNTS = 3	Full-time = 131 Part-time = 25 Various = 6 Missing = 4	East of England = 14 London = 20 Midlands = 37 North East = 30 North West = 23 South East = 21 South West = 17 Missing = 4	Primary care = 22 Community care = 20 Secondary care = 43 Older people's care = 7 Mental health = 18 Learning disability = 2 Adult general/medical/surgical = 38 Critical care = 16

Late-career	[1963–2023]	[51–71+]	Female = 1274 Male = 133 PNTS = 16	White = 1280 BME = 109 PNTS = 34	Full-time = 590 Part-time = 614 Various = 119 Missing = 100	East of England = 125 London = 147 Midlands = 291 North East = 219 North West = 219 South East = 201 South West = 201 Missing = 20	Primary care = 229 Community care = 200 Secondary care = 503 Older people's care = 71 Mental health = 111 Learning disability = 20 Rehabilitation/longer-term care = 28 Adult general/medical/surgical = 155 Critical care = 79 Other = 27

Abbreviation: PNTS, Prefer not to say.

## Data Availability

The data that support the findings of this study are available from the corresponding author upon reasonable request.

## Supporting Information

Supporting Information 1 Supporting File 1—Consolidated Criteria for Reporting Qualitative Studies (COREQ) checklist for qualitative research [27].

Supporting Information 2 Supporting File 2—Semistructured discussion guides for early- and late-career nurses.

## References

[1] WHO (2024). *Nursing and Midwifery*.

[2] American Association of Colleges of Nursing (2019). *Nursing Shortage*.

[3] Danish Nurses Organization (2018). *Nursing Retention Will be Studied*.

[4] Rcn (2023). *Dangerously Inadequate Staffing Levels: NHS Waiting List Growing 4 Times Faster Than Nurse Workforce*.

[5] World Health Organisation (2023). Nursing and Midwifery.

[6] Shembavnekar N., Buchan J., Bazeer N. (2022). *NHS Workforce Projections 2022*.

[7] Kelly E., Stoye G., Warner M. (2022). *Factors Associated With Staff Retention in the NHS Acute Sector*.

[8] NHS Digital (2022). *HCHS Nurses and Health Visitors Turnover by Age and Ethnicity*.

[9] Stockton I. (2021). *Nurses Leaving the NHS Acute and Community Sectors (PDF)*.

[10] Ball J. E., Griffiths P. (2022). Consensus Development Project (CDP): An Overview of Staffing for Safe and Effective Nursing Care. *Nursing Open*.

[11] Nilasari P., Hariyati R. T. S. (2021). Systematic Review of Missed Nursing Care or Nursing Care Left Undone. *Enfermeria Clinica*.

[12] NMC (2023). *Leavers’ Survey 2022*.

[13] Dall’Ora C., Ejebu O., Ball J., Griffiths P. (2023). Shift Work Characteristics and Burnout Among Nurses: Cross-Sectional Survey. *Occupational Medicine*.

[14] ICN (2022). Alarming Increase in Industrial Action by Nurses is a Symptom of a Global Crisis in Healthcare Systems.

[15] Buchan J., Catton H. (2023). Recover to Rebuild: Investing in the Nursing Workforce for Health System Effectiveness. *International Council of Nurses*.

[16] Ejebu O., Philippou J., Turnbull J. (2024). Coming and Going: A Narrative Review Exploring the Push-Pull Factors During Nurses’ Careers. *International Journal of Nursing Studies*.

[17] Sasso L., Bagnasco A., Catania G. (2019). Push and Pull Factors of Nurses’ Intention to Leave. *Journal of Nursing Management*.

[18] Philippou J. (2015). Employers’ and Employees’ Views on Responsibilities for Career Management in Nursing: A Cross‐Sectional Survey. *Journal of Advanced Nursing*.

[19] NHS England (2023). NHS Long Term Workforce Plan.

[20] NHS England (2021). Our NHS People Promise.

[21] Conservative Party (2019). Conservative Party Election Manifesto.

[22] Ejebu O., Turnbull J., Atherton I. (2024). What Might Make Nurses Stay? A Protocol for Discrete Choice Experiments to Understand NHS Nurses’ Preferences at Early-Career and Late-Career Stages. *BMJ Open*.

[23] Kaewpan W., Peltzer K. (2019). Nurses’ Intention to Work After Retirement, Work Ability and Perceptions After Retirement: A Scoping Review. *The Pan African medical journal*.

[24] Pressley C., Garside J. (2023). Safeguarding the Retention of Nurses: A Systematic Review on Determinants of Nurse’s Intentions to Stay. *Nursing Open*.

[25] Cleaver K., Markowski M., Wels J. (2022). Factors Influencing Older Nurses’ Decision Making Around the Timing of Retirement: An Explorative Mixed-Method Study. *Journal of Nursing Management*.

[26] Ball J., Turnbull J., Ejebu O. (2025). Dataset Supporting an Article ‘Employers’ Offers to Recruit and Retain Nurses in England: A Cross-Sectional Study. *N50k Evaluation Study*.

[27] Tong A., Sainsbury P., Craig J. (2007). Consolidated Criteria for Reporting Qualitative Research (COREQ): A 32-Item Checklist for Interviews and Focus Groups. *International Journal for Quality in Health Care*.

[28] Ball J., Ejebu O., Saville C. (2022). What Keeps Nurses in Nursing? A Scoping Review Into Nurse Retention. *Nursing Times*.

[29] Malterud K., Siersma V. D., Guassora A. D. (2016). Sample Size in Qualitative Interview Studies: Guided by Information Power. *Qualitative Health Research*.

[30] Markowski M., Cleaver K., Weldon S. M. (2020). An Integrative Review of the Factors Influencing Older Nurses’ Timing of Retirement. *Journal of Advanced Nursing*.

[31] Montayre J., Knaggs G., Harris C. (2023). What Interventions and Programmes Are Available to Support Older Nurses in the Workplace? A Literature Review of Available Evidence. *International Journal of Nursing Studies*.

[32] Uthaman T., Chua T. L., Ang S. Y. (2016). Older Nurses: A Literature Review on Challenges, Factors in Early Retirement and Workforce Retention. *Proceedings of Singapore Healthcare*.

[33] Viviani C., Bravo G., Lavallière M. (2021). Productivity in Older Versus Younger Workers: a Systematic Literature Review. *Work*.

[34] Vázquez‐Calatayud M., Eseverri‐Azcoiti M. C. (2023). Retention of Newly Graduated Registered Nurses in the Hospital Setting: A Systematic Review. *Journal of Clinical Nursing*.

[35] Karnieli-Miller O., Strier R., Pessach L. (2009). Power Relations in Qualitative Research. *Qualitative Health Research*.

[36] Lumivero (2024). *Nvivo*.

[37] Braun V., Clarke V. (2006). Using Thematic Analysis in Psychology. *Qualitative Research in Psychology*.

[38] NHS England Digital (2025). NHS Workforce Statistics.

[39] Al Yahyaei A., Hewison A., Efstathiou N., Carrick-Sen D. (2022). Nurses’ Intention to Stay in the Work Environment in Acute Healthcare: A Systematic Review. *Journal of Research in Nursing*.

[40] Ball J., Palmer W., Ejebu O. (2022). *Evaluation of the N50K Programme*.

[41] Ball J., Ejebu O. (2021). *Retention of NHS Nurses: Scoping Review. Report for NHSEI*.

[42] Brown J., McDonald M., Besse C. (2021). Nursing Students’ Academic Success Factors: A Quantitative Cross-Sectional Study. *Nurse Educator*.

[43] Kallio H., Kangasniemi M., Hult M. (2022). Registered Nurses’ Perceptions of Having a Calling to Nursing: A Mixed‐Method Study. *Journal of Advanced Nursing*.

[44] Roth C., Wensing M., Breckner A., Mahler C., Krug K., Berger S. (2022). Keeping Nurses in Nursing: A Qualitative Study of German Nurses’ Perceptions of Push and Pull Factors to Leave or Stay in the Profession. *BMC Nursing*.

[45] Majeed N., Jamshed S. (2021). Nursing Turnover Intentions: the Role of Leader Emotional Intelligence and Team Culture. *Journal of Nursing Management*.

[46] Marufu T. C., Collins A., Vargas L., Gillespie L., Almghairbi D. (2021). Factors Influencing Retention Among Hospital Nurses: Systematic Review. *British Journal of Nursing*.

[47] Hörberg A., Gadolin C., Skyvell Nilsson M., Gustavsson P., Rudman A. (2023). Experienced Nurses’ Motivation, Intention to Leave, and Reasons for Turnover: A Qualitative Survey Study. *Journal of Nursing Management*.

[48] Palmer W., Leone C., Appleby J., Trust N. (2021). *Recruitment of Nurses from Overseas: Exploring the Factors Affecting Levels of International Recruitment*.

