# Supplementary material for: Values and Workplace Expectations to Facilitate Retention: Perspectives From Nurses at Two Ends of the Career Spectrum
Source: J Nurs Manag. 2025 Sep 12;2025:9912825. doi: 10.1155/jonm/9912825 (PMC12449091; doi:10.1155/jonm/9912825)
Supplement: Supporting Information 2 — Supporting File 2—Semistructured discussion guides for early- and late-career nurses. [file 9912825.f2.docx]

**Supplementary file**

**Semi-structured focus group (and interview) guide for early-career nurses**

**Round of introduction**

- Name, job title, geographical location, year of graduation

**Pull-push factors**

- What do you value most about your profession?
- Which job factors influence your decision to stay in nursing?
- Which aspects of your daily work keep you practising?
- What does your workplace/NHS do best?
- Which aspects of your daily work would need to be improved?
- Have you considered/are you considering leaving your profession? When and why?
- What would need to change (at work) to prevent you from leaving? / Why did you change your mind about not leaving the profession?

**N50K, Covid and retention**

- Have you heard about the N50K programme?
  - Which role (if any) has this programme played in your decisions to stay/leave your profession?
- How has Covid-19 influenced your feelings about leaving or staying in your job/ nursing?

**Push-pull factors**

I’d like to know more about the different factors in your work environment/job that might influence your decisions to stay or leave your current job, or the NHS more generally. Can you tell me about the following job factor, how satisfied are you with it and what role in your decision to leave/stay in your profession?

- Mentoring/preceptorship to help with the transition period (from student to nurse) at the beginning of your career?
  - If yes, how satisfied were you with your scheme?
- If not, would you have benefited from having a mentoring scheme?
- Relationship with your (NHS) manager
- Relationship with your colleagues
- Staffing levels and equipment
- Patient care
- Workload
- Working environment (facilities (e.g. rest facilities, staff-room), technology/IT)
- Opportunities for career development
- Autonomy and your role in decision-making
- Contract and remuneration
- Working hours/flexible working

Are there any other aspects that you think we have not addressed?

**Semi-structured focus group (and interview) guide for late-career nurses**

**Round of introduction**

- Name, job title, geographical location

**Retirement decisions**

- Have you given any thought about when/at what age you might retire?
- Are you aware of your workplace policies on working after retirement?
- Do you plan to work as a nurse after retirement?
- What could your workplace do to incentivise you to work after retirement?
- What (job) factors do you consider as a barrier or support to work after retirement?

**N50K, Covid and retirement plans**

- Have you heard of the N50K programme?
  - Which role (if any) has it played in your retirement plans?
- Has Covid-19 played any role in your retirement plans?

**Push-pull factors**

I’d like to know more about the different factors in your work environment/job that might influence your decisions to stay or leave your current job, or the NHS more generally. Can you tell me about the following job factor, how satisfied you are with it and whether it has influenced your decision to retire?

- Working environment (e.g. facilities, technological changes)
- Working conditions (e.g., flexibility, autonomy in choosing shifts, etc.)
- Workload
- Relationship with your (NHS) manager and non-clinical managers
- Relationship with your colleagues (e.g. presence of workplace incivility/bullying)
- Opportunities for career development
- Opportunities to mentor other (and younger) colleagues
- Recognition from managers/colleagues
- Contract and remuneration.
  - Do you feel that your salary reflects your expertise?

Are there any other aspects that you think we have not addressed?
